# Graphene preparation and graphite exfoliation

**DOI:** 10.3906/kim-2101-19

**Published:** 2021-06-30

**Authors:** Ahmed A. MOOSA, Mayyadah S. ABED

**Affiliations:** 1 Materials Engineering Technology Department, Engineering Technical College, Middle Technical University, Baghdad Iraq; 2 Department of Materials Engineering, University of Technology, Baghdad Iraq

**Keywords:** Graphene, graphene oxide, graphite, exfoliation

## Abstract

The synthesis of Graphene is critical to achieving its functions in practical applications. Different methods have been used to synthesis graphene, but graphite exfoliation is considered the simplest way to produce graphene and graphene oxide. In general, controlling the synthesis conditions to achieving the optimum yield, keeping the pristine structure to realize on-demand properties, minimum layers with the smallest lateral size, and minimum oxygen content are the most obstacles experienced by researchers. Each application requires a specific graphene model, graphene oxides GO, or even graphene intercalated compounds (GIC) depending on synthesis conditions and approach. This paper reviewed and summarized the most researches in this field and focusing on exfoliation methods.

## 1. Introduction

It is known that graphite and diamond are the two natural crystalline allotropic forms of carbon for a prolonged time. The atomic arrangement of carbon atoms in these materials gives different properties. For example, graphite is soft and black, and stable, while diamond is hard and transparent [1]. Diamond and graphite consist of extended networks of sp^3^- and sp^2^ -hybridized carbon atoms, respectively [2]. 

Allotropes are elements that are chemically identical but vary in their physical properties. In 1985, the discovery of fullerenes by Kroto et al. [3] marked the beginning of an era of synthetic carbon allotropes with striking properties. Iijima, in 1991, discovered carbon nanotube (CNT) [4]. While in 2004, graphene, which is a two-dimensional form of graphite, was isolated at Manchester University by Novoselov et al. [5]. 

Graphene is a 2-dimensional structure consists of a single atom thick sheet of carbon crystallized as a honeycomb structure monolayer [6]. Graphene has been considered as the fundamental building block for all sp^2^ graphitic materials, including (0D) fullerenes, (1D) carbon nanotubes, and stacked into (3D) graphite [7].

## 2. Allotropes of carbon

**Figure 1:**
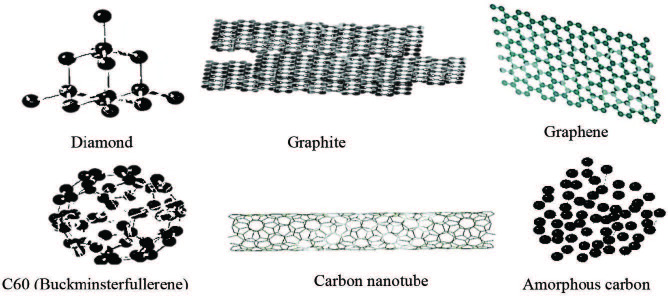
Diamond, graphite, graphene, C60 (Buckminsterfullerene), carbon nanotube, and amorphous carbon are graphical examples of carbon allotropes.

Allotropes of carbon can be defined by valence bonds hybridizations sp^n^ (n = 3 for a diamond, and n = 2 for graphite). The physical, chemical and mechanical properties of the allotropes of carbon (see Figure 1) are different depending on valence bond hybridization [8].

Graphite has a layered planar structure wherein each carbon atoms layer is arranged in a honeycomb lattice with a separation of 0.142 nm. The interplanar spacing, d, is (0.335nm) [9]. In diamond, the carbon atoms are arranged in a face-centered cubic crystal structure (FCC) system with diamond lattice [10]. Fullerenes are zero-dimensional hollow carbon cage structures with carbon atoms arranged in a lattice similar to the soccer-ball. The most widely publicized fullerene is buckyball (C60). Carbon nanotubes (CNTs) are one dimension structure that can be viewed as a hollow cylinder formed by rolling a two-dimensional graphene sheet into a cylinder with a half fullerene at its ends. Carbon nanotubes are either single-walled CNTs (SWNT) or multiwalled CNTs (MWNT) [11]. Graphene is a flat single atom thick sheet of carbon packed into a honeycomb crystal plane and has a 2D structure [6]. 

## 3. Graphene

Due to the outstanding features and large applications, Graphene has attracted great interest from scientists and engineers [7]. Moreover, it is the thinnest and strongest material that is ever subjected to measurement [12]. Graphite is considered to consist of huge numbers of graphene layers. There are two kinds of bonds form among the graphene layers. The graphene layers are held together by the weak Vander Waal attraction with a length of about 0.341nm between the adjacent graphene layers [13]. This weak attraction between layers can ease the exfoliation into individual layers. The other bond is between carbon-carbon atoms with 0.142 nm spacing, which is a strong covalent bond entire each layer [14].

 Graphenes can exist in different forms: graphene oxide, chemically modified graphenes, bilayer graphenes, etc. These nanomaterials have many medical applications, integrated circuits, transparent conducting electrodes, desalination, solar cells, energy storage, biodevices, etc. [15].

Due to its unique thermal, electrical, and mechanical characteristics, Graphene has been utilized in many applications. A Single-layer of graphene has thermal conductivity as high as 5000 W/m.K [16] and higher about 10 times than Cu and superior to carbon nanotubes (CNTs) and diamond [17]. Graphene has high mobility of charge carriers (200000 cm^2^/V.S) [18], which means an electron moves through it without much scattering or resistance. Graphene has a high specific surface area of 2630 m^2^/g [19]. The unique structure enables graphene of many distinctive properties and promising applications. Graphene is the strongest material ever measured and has excellent mechanical properties with an elastic modulus of 1 TPa and ultimate tensile strength of 130 GPa [20]. Thus, graphene is the strongest material ever discovered to date. It is estimated that graphene is 100 times stronger than the best steel ever for the same thickness [17]. Thus, graphene is a promising material for high-performance nanoelectronics, heat dissipation, sensors, field emission, and transparent conductor. One single-layer of graphene has a transparency of 97.7% of incident light and absorbs only 2.3% and, it is almost completely transparent [21]. 

Graphene is treated nowadays as a rising new material in the field of high-efficiency sensors [22], fuel cells [19,23], renewable energy sources [24,25], transparent electrodes [26], and nanocomposite materials [27-29]. Many methods are used to synthesis graphene such as micromechanical cleavage [5], chemical vapour deposition [30], and [31], the epitaxial growth on silicon carbide [32], liquid-phase exfoliation [33,34] and graphene oxide reduction by different reducing agents [18,35,36], and graphite intercalation [37].

Epitaxial growth and chemical vapor deposition are used to prepare graphene with excellent high-value optoelectronic and electronic applications. However, the high costs and difficulties encountered in chemical vapor deposition and epitaxial growth limit their applicability. Wet chemical graphite exfoliation routes include graphite oxide exfoliation, liquid-phase exfoliation (LPE), and electrochemical are the most commonly used methods.

## 4. Graphene synthesis 

4.1. Chemical vapour deposition (CVD)

**Figure 2:**
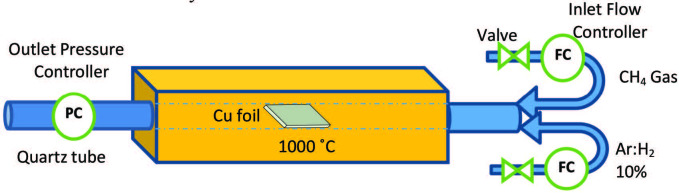
Chemical vapor deposition (CVD) process.

The chemical vapor deposition (CVD) on transition metals (e.g., Fe, Co, Ni, Cu, Pd) is another method used to prepare graphene, which requires a temperature as high as 1000 °C with a hydrocarbon gas flow as precursor [30,31,38]. This method is applied at high temperatures when exposing transition metal to low concentration hydrocarbon gas till the surface is saturated with carbon atoms [22,39] (see Figure 2).

Methane gas is generally used as a source of carbon. Hydrogen and argon are also used with methane as reaction stabilizers and enhancing the film uniformity. Zhan et al. in 2018 [40] showed a strong correlation between substrate morphology and graphene domain density. The use of a pre-annealed Cu foil followed by electro-polishing treatment will give ultra-smooth Cu surfaces. The graphene grows on annealed electro-polished Cu substrates show better quality in terms of lower domain density and higher layer uniformity than those grown on Cu substrates with only annealing or only electro-polishing treatment. 

Well-ordered graphite films of nanometer thickness have been grown on Ni substrates using chemical vapor deposition (CVD) with a thickness of a few graphene layers with graphite film thickness is about 1.5 ± 0.5 nm [41].

Ni as the catalyst has some limitations such that an over a small region of few to tens of microns, single and few-layered graphene is obtained and not homogeneously throughout the entire substrate. The control lack over the number of the layers is partial because, upon cooling, the carbon segregation from the metal carbide happens rapidly within the nickel grains and heterogeneously at the grain boundaries. 

The use of polycrystalline copper foils as a substrate in CVD gave exceptional results in uniform deposition of high-quality single-layered graphene over large areas [30]. This method can produce high-quality graphene and suitable for mass production, but it is not cost-effective and easy. 

### 4.2. Epitaxial growth (SiC heating)

**Figure 3:**
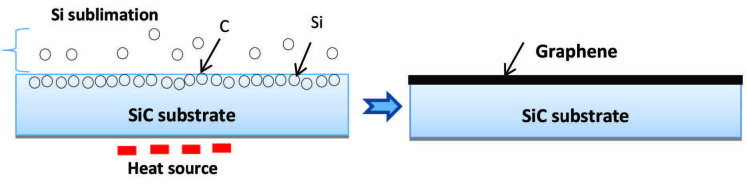
Epitaxial growth of Graphene (SiC heating) [43].

The availability of high-quality and large-area graphene is a crucial requirement for the production of graphene-based nanoelectronics. As discussed above, the other methods for graphene production have their limitation. The CVD method [38] requires a purification process to eliminate the catalyst particles and transfer Graphene to another substrate; the chemical reduction of GO requires chemicals, which can affect graphene properties; the scotch tape method [5] is not suitable for large scale production of Graphene. The field of epitaxial graphene on silicon carbide is rapidly growing by annealing SiC as an insulator at high temperatures under vacuum to form a graphitic surface by sublimating Si atoms [42] (see Figure 3) [43]. The potential of epitaxial graphene as new electronic material is now well recognized [44]. Silicon carbide (SiC) was used as substrates for the epitaxial growth of graphene. The substrate is heated at high temperatures (>1250 °C) and low pressures (~10−6 Torr). Ultrathin epitaxial graphite films were grown by thermal decomposition on the (0001) surface of 6H-SiC. The films are typically composed of 3 graphene sheets [45]. The graphene epitaxial growth on silicon carbide is considered an ideal material for high-end electronics that might surpass silicon in terms of key parameters such as speed, feature size, and power consumption [44]. The epitaxial growth method can prepare high-quality graphene with excellent properties, but it is not easy to obtain a high production rate with this method.

### 4.3. Arc discharge 

**Figure 4:**
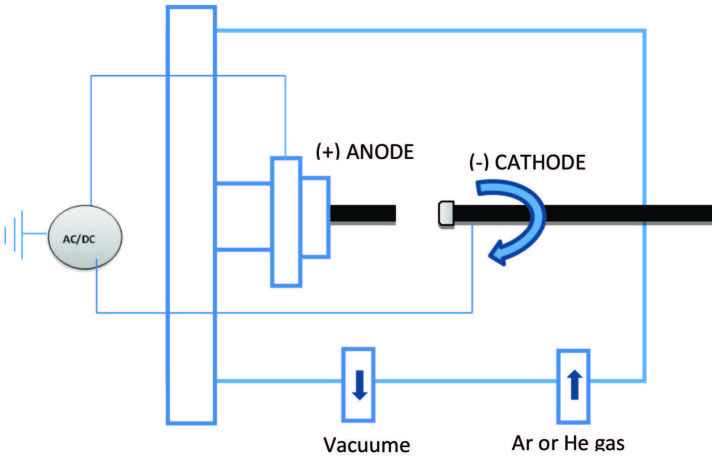
Arc discharge apparatus.

Kretschmer et al. [46] were the first that utilized the arc-discharge method to synthesize C60 [47]. The main concept of graphene synthesis by arc discharge is similar when applying an electric field between two graphite electrodes under argon or helium gases. When the electrodes’ distance between the two electrodes becomes close to 1–2 mm, the arc discharge starts, and the evaporated materials from the anode are deposited on the cathode. The anode is almost previously compounded with catalysts (Ni, Fe, and B). Also, the cathode should be rotated to keep the distance constant [48]. The arc discharge process is illustrated in Figure 4.

### 4.4. Unzipping of carbon nanotubes

**Figure 5:**
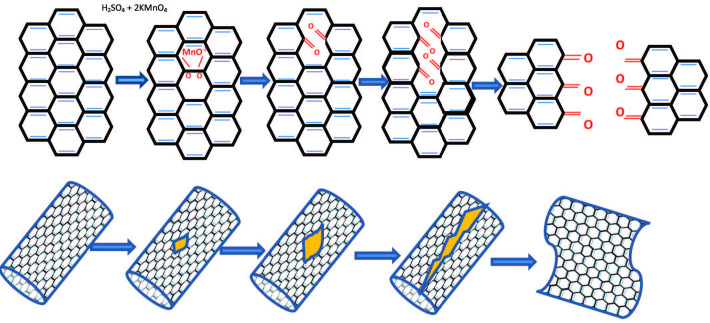
Carbon nanotubes unzipping mechanisim [50].

Saito et al. (2002) showed that MWCNTs could be cut to several nanometers of CNTs by sonication in mixed strong acids (H_2_SO_4 _(90%) /HNO_3 _(70%) [49]. In comparison, Kosynkin et al. (2009) obtained oxidized graphene nanoribbons by suspending MWCNTs in concentrated H_2_SO_4_ and then treated with KMnO_4_. The isolated graphene nanoribbons were highly soluble in water, ethanol, and other polar organic solvents [50]. The nanotubes’ unzipping seems similar to the ‘unzipping’ of graphite oxide [51] and [52]. The opening could occur longitudinally or spirally way, depending on the attacked site and chirality angle. Manganate ester formation (O=MnO^-2^=O) is the main reason to destroy the alkene structure of CNT, making these initial attacked sites more exposable to the next intensive reaction with permanganate [50]. Figure 5 illustrates the proposed unzipping mechanism of CNTs [50]. 

### 4.5. Graphene synthesis by oxidation of graphite

4.5.1. Graphene oxide

**Figure 6:**
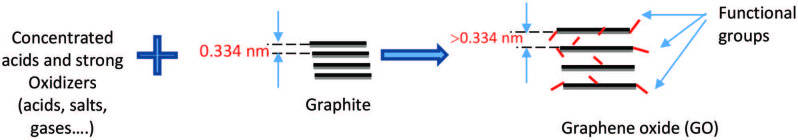
Oxidation of graphite.

Large amounts of graphene can be produced at a reasonable cost from graphite oxide. Graphene oxide can be prepared by oxidation of graphite using concentrated acids and strong oxidants (Figure 6). The oxidation of graphite to form graphite oxide was introduced in 1859 by Brodie [53] when graphite was oxidized in the presence of potassium chlorate KClO_3_ and fuming nitric HNO_3_ + NO_2_. Staudenmaier developed this method later in 1898 [54]. Another improvement in the Brodie method was made by Hofmann and König (1937) [55]. Hummers and Offeman made further development on the Brodie method [56]. Marcano et al. [57] improved on the Hummers method by eliminating sodium nitrate, thus excluding toxic nitrous gas production.

In these modifications, the key developments concentrate on the use of safer oxidants and more effective procedures. Staudenmaier and Hofmann’s methods use potassium chlorate, and the methods of Hummers and Tour use potassium permanganate as the primary oxidant. Nitric, sulphuric and phosphoric acids or their mixtures are used as solvents in both synthesis methods.

Brodie was the first to prepare graphite oxide by oxidation of graphite in a mixture of potassium chlorate. An oxidizing agent and nitric acid helped to intercalate graphite and expand it [53]. Later on, Staudenmaier et al. [54] used nitric acid: sulphuric acid with a volume ratio of 1:4 in an ice bath. Then, graphite was added (4.37 g for every 100 mL) with continuous stirring. The oxidizing agent is potassium chlorate. The weight ratio of graphite to the oxidizing agent is 1:11. Staudenmaier’s method is considered safer than the Brodie method, but toxic gasses are still created; this is why using the Hummers method more than Staudenmaier and Brodie method. In 1958, Hummer developed a new method [56], which is the most common method for preparing graphene oxide using KMnO_4_, NaNO_3_, and H_2_SO_4_ to oxidize graphite into graphite oxide within a few hours. The advantages of Hummer’s method are as follows. First, the reaction is completed within a few hours. Second, the oxidizing agent KMnO_4_ is used to replace KClO_3_ and improving reaction safety by avoiding the evolution of explosive ClO_2_ and KClO_3_. Third, the use of NaNO_3_ to replace HNO_3_ eliminates the forming of acid fog [58].

Later on, a new method was developed by Tour and his group in 2010 to improve the Hummers method in the degree of GO oxidation as well as to minimize the generation of toxic gases (NO_2_, N_2_O_4_). In this method, sodium nitrate (NaNO_3_) was replaced by potassium permanganate. The mixture of reactions consists of (9:1) H_2_SO_4_: H_3_PO_4_ for the oxidation reaction [57].

Graphene oxide is a single layer of graphite oxide. The chemical oxidation of graphite and its subsequent exfoliation in water results in single-layered GO sheets [59]. Graphene oxide contains a mixture of sp^2-^ and sp^3-^ hybridized carbon (C) atoms decorated with oxygen-containing functionalities. These functional groups include hydroxyl (C–OH) and epoxide (C–O–C) functional groups on the basal plane and carbonyl (C=O)) and carboxyl (COOH) groups at the sheet edges [60]. These functionality groups introduce sp^3^ defect sites to the nanosheets, distorting the intrinsic conjugated π system and lowering overall strength and conductivity. The saturated sp^3 ^carbon atoms bound to oxygen make GO an insulator. Graphene oxide is an electronically hybrid substance that displays both a large energy gap between the σ-states of its sp^3-^ bonded carbons and conducting π-states from sp^2^ carbon sites [61]. By reducing chemistry, the tunability of the ratio of the sp^2^ and sp^3^ fractions is a powerful way to tune its bandgap and, thus, controllably transform graphene oxide from an insulator to a semiconductor or semi-metal [62].

#### 4.5.2. Reduction of graphene oxide 

With the recovery of a conjugated structure, graphene oxide can be reduced to graphene by eliminating the oxygen-containing groups. Reduced GO (rGO) sheets are generally referred to as chemically modified graphene, chemically transformed graphene, or reduced graphene oxide [62].

**Figure 7:**
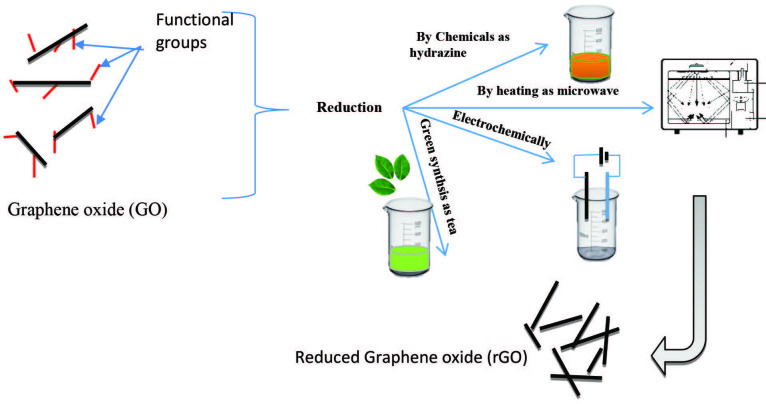
Reduction of graphene oxide.

Graphene oxide is an electrically insulating material but can be converted to a conductor by chemical reduction process, electrochemical reduction, thermal treatment, and green reduction [63,64,65], as shown in Figure 7. However, none of the reported reduction methods yield complete oxygen removal, resulting in only partial restoration of the sp^2^ conjugated graphene network [62]. In thermal or chemical reduction, the functional groups C=O and O=C-OH could be partially removed or converted to a new chemical species such as (C-OH). The degradation of the electrical properties of the RGO is due to the remaining functional groups C-OH or C-O-C in RGO that could not be reduced easily [66].

##### 4.5.2.1. Thermal reduction

Thermal reduction of GO was made by conventional heating (thermal annealing) [67], microwave [68], and photo-reduction [69]. In thermal annealing, GO is heated to a high temperature under inert gases such as argon gas. The rapid increase in temperature makes oxygen functional groups decompose into CO and CO_2_ gases. These gases create large pressure between graphene oxide stacked layers, which are enough to exfoliate individual GO layers is enough to separate two stacked GO sheets [67].

Microwave irradiation (MWI) is another thermal reduction method of GO using a microwave oven. This is a rapid method and gives uniform heating of the GO [68]. Zhang et al. proposed another method: a photo-reduction of GO using femtosecond laser irradiation [69].

##### 4.5.2.2. Chemical reduction 

Another method of GO reduction is the chemical method, which can be carried out using a chemical reducing agent. This is an effective method for mass production of graphene because of availability and low cost compared with other reduction methods [65]. Stankovich et al. were the first to prepare reduced graphene oxide (rGO) [36] using hydrazine as a chemical reducing agent. Hydrazine and its derivatives, such as hydrazine hydrate (NH_2_NH_2_.xH_2_O) and dimethyl-hydrazine, can be used as a chemical reducing agent for GO.

In organic chemistry, metal hydrides have been accepted as strongly reducing reagents such as sodium borohydride, lithium aluminum hydride, and sodium hydride. These reducers, unfortunately, have a good response to aqueous solutions [70]. S. Some et al. utilized thiophene as a reducing agent of graphene oxide (GO) by donation of electrons with acceptance of oxygen while it was converted into an intermediate oxidized polymerized thiophene that losses sulfur atoms and transformed into polyhydrocarbon that heat-treated to obtained reduced GO [71]. M. J. Fernandez-Merino et al. used different reducing agents sodium borohydride, pyrogallol, and vitamin C, in addition to hydrazine and made a comparison among them. They found that vitamin C has highest yield suspension compared with other compounds [72]. T.T. Dang et al. reported the influence of temperature on the reduction of graphene oxide by hydrazine in N, N-dimethylformamide (DMF)/H_2_O and the dispersibility of the resultant graphene in DMF. The results showed highly reduced GO with a high C/O ratio and good dispersibility in DMF which diminishes the formation of irreversible graphene sheet aggregates. So, this dispersibility of GO was 1.66 to 0.38 mg/mL when the reduction temperature increased from 25–80 °C [73].

R. Tarcan et al. reduced graphene oxide in N, N-dimethylformamide without using any reducing agents using the solvothermal microwave-assisted method. So, this method gives stable and highly concentrated dispersions of GO through partial removal of oxygenated groups from graphene oxide surface [74].

##### 4.5.2.3. Electrochemical reduction

The electrochemical reduction of GO relies on removing oxygen functional groups using regular electrochemical cells containing aqueous solutions (electrolyte) at ambient temperature [75,76]. Graphene oxide reduction occurs by the exchange of electrons between the inert electrodes and GO sheets or films. This method eliminates the usage of dangerous reducing agents as hydrazine.

##### 4.5.2.4. Green synthesis

The green synthesis approach of graphene reduction is rapidly improved due to its low cost, ease of handling. It is an eco-friendly method without hazards, harsh, and expensive materials [77]. 

Biomolecules, microbes, and plant extracts are used to reduce and cap agents widely used to synthesize metal nanoparticles. Generally, plants contain a wide variety of bio-molecular substances, including amino acids, proteins, alcoholics, polysaccharides, enzymes, polyphenols, and vitamins. Plant extracts may also be used for graph synthesis as a reduction and stabilization agent [78,79]. Ascorbic acid or (Vitamin C) is a recently reported reducing reagent for graphene oxide with benefits over hydrazine [72]. Ascorbic acid is nontoxic and has higher chemical stability with water so that the produced RGO does not result in aggregation. Taking into consideration the environment and human health in recent years, reduced graphene oxides (RGOs) were produced by green reduction of graphene oxide (GO) using ascorbic acid [72], glucose [80], fructose and sucrose [68], tea leaves [77], melatonin [79] and alcohols [81].

### 4.6. Mechanical exfoliation of graphite

**Figure 8:**
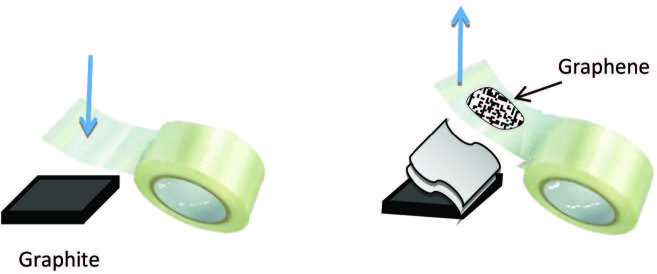
Scotch tape method for graphite exfoliation [82].

Geim and Novoselov [7] prepared a single graphene sheet by peeling off a sheet of graphite using Scotch tape (see Figure 8) [82]. This method involves repeatedly peeling highly oriented pyrolytic graphite (HOPG) using scotch tape. The process has been optimized to produce single-layer graphene (SLG) with high structural quality and more than 100 μm^2^ in size [83]. This method is called the Scotch tape approach, which is not suitable for large production of graphene.

### 4.7. Liquid-phase exfoliation (LPE)

Layered materials such as graphite consist of two-dimensional platelets weakly stacked to form three-dimensional structures. In graphite, these layers form strong chemical bonds in-plane but display weak out-of-plane bonding. This facilitates the exfoliation process to form so-called nanosheets (about micrometers wide but less than a nanometer thick). Such exfoliation leads to an increase in the material’s surface area, over 1000 (m^2^/g), and enhanced surface activity [84]. The increase in the surface area of material can enhance their chemical and physical reactivity. This is very vital in many applications where the surface activity is important such as catalytic materials, ion exchange [85], fillers materials in composites [86], intumescent (or thermally expanded) material [84].

The graphene preparation by reducing graphene oxide is the most popular scalable method, but the RGO produced has many structural defects and residual oxygen from the strong oxidation steps. These defects disrupt the band structure, resulting in the deterioration of mechanical, electrical, and thermal properties, making graphene unique [36,62].

To minimize oxide defects in the graphene sheet, many attempts were made to exfoliate graphite into a liquid solution to obtain colloidal suspension of graphene layers in the solution. 

Liquid-phase exfoliation (LPE) is a top-down method of exfoliation of natural graphite into few-layer graphenes dispersed in liquid media [87] and are becoming more desirable because they are cost-efficient, using inexpensive graphite, scalable process, produce defect-free graphene, and can be used to deposit the dispersed graphene in a variety of environments and on different substrates not available in using mechanical cleavage or growth methods [88–90].

This process involves graphite dispersal in a solvent, ultrasound exfoliation or other mechanical dispersal procedures, and purification. The involved mechanisms of fragmentation and exfoliation have usually been attributed to the force induced by ultrasound and the interaction with the solvent molecules [33,34,88,91,92]. Liquid phase exfoliation is a four-step method as illustrated by Shen and collaborators [93–95] that include: 1) graphite immersion into the liquid medium, 2) intercalation of the graphite, 3) the exfoliation of these planes, and 4) the stabilization of the exfoliated materials isolated in the medium. In step one, the liquid medium for graphite immersion should effectively reduce van der Waals forces’ attraction between the layers of the nonexfoliated material. The liquid molecules must be inserted between the layers of the crystal before exfoliation. During the intercalation step of the graphite, the interaction between the graphite graphene planes is decreased. In the third step, the exfoliation of these planes occurs, which can be mechanically assisted or not. When 2D material with the liquid molecules inserted is exposed to a mechanical fragmentation force such as shear, van der Waals’ attractive forces can be overcome, facilitating the individual layers’ exfoliation. Finally, in the fourth step, the exfoliated material isolated in the liquid medium should be remaining stable. A fundamental characteristic of this method is the possibility of producing mono and few-layer graphenes stabilized in different media [93–95]. 

**Figure 9:**
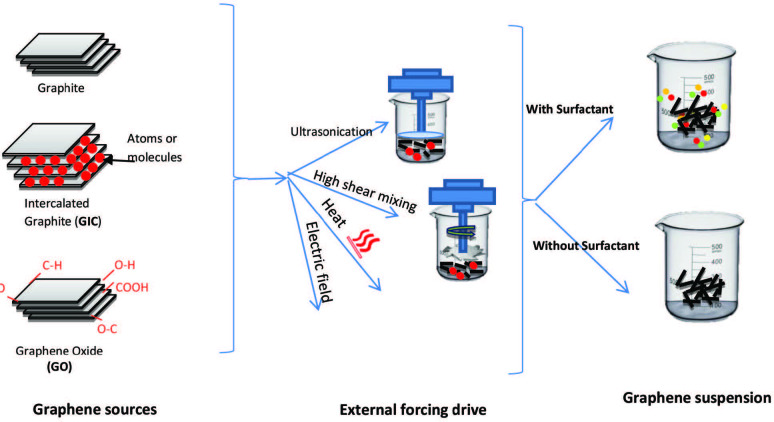
Liquid phase exfoliation.

Different precursors of graphitic material are used in Liquid-phase exfoliation, such as graphite oxide, natural graphite, graphite intercalation compound (GIC), and sonication-free liquid-phase exfoliation. These graphite precursors are directly subjected to a solvent treatment to weaken the van der Waals attractive forces between graphene interlayers using external driving forces such as ultrasonication, electric field, shearing, or heat (see Figure 9) [96].

#### 4.7.1. Liquid-phase exfoliation of graphite oxide

Liquid-phase exfoliation of graphite oxide is the most widely used method for the preparation of graphene. In this method, the graphite is intercalated with strong oxidizing agents [53,54,56], followed by graphite layers’ expansion via sonication. The reduction of the obtained graphene oxide to graphene is usually conducted by either thermal or chemical approaches [36]. 

To exfoliate, it is important to resolve the attractions of the van der Waals between adjacent layers. One way to reduce these attractions by oxidation and chemical intercalating reactions is to increase the distance between adjacent layers [26,97,98].

The oxygen functional groups such as hydroxyl and epoxy are inserted and attached to graphite layers during graphite oxidation. The d-spacing between these layers has increased significantly from 3.4 to around 7.0 A [64,67].

The hydroxyl or carboxyl groups covalently bonded with graphene oxide make graphene oxide dispersable in some organic solvents at concentrations of up to 1 mg/mL [36] and water up to 7 mg/ mL [99]. However, the oxidization process significantly causes defects in graphene. Stankovich et al. [36] used hydrazine to reduce GO, and the chemically reduced graphene product (CRG) still has some defects. Other reduction methods are as follows: the solvothermal reduction method of GO in N, N-dimethylformamide (DMF) with hydrazine [100] or without hydrazine [101] as a reducing agent, the use of metal Fe to reduce exfoliated GO [102]. None of these reduction methods can recover the graphene structure completely, and some oxygen-containing groups are still irremovable.

#### 4.7.2. Liquid-phase exfoliation of natural graphite

The liquid phase exfoliation method and exfoliation of GO of graphite are technically similar, but the first does not involve the oxidation step. Graphene flakes can be produced by exfoliation of graphite using chemical wet dispersion followed by ultrasonication in water and organic solvents. 

This method’s principal attraction is a simple and scalable process where pristine graphite or expandable graphite is subjected to a solvent treatment to weaken the van der Waals attractive forces between graphene interlayers. External driving forces such as ultrasonication, shearing, or electric field can be applied to help exfoliation into graphene [92]. Graphene’s exfoliation occurs because of the strong interactions between the solvent molecules and the graphitic basal planes, overcoming the energetic penalty for exfoliation and subsequent dispersion. For successful exfoliation, overcoming the van der Waals attractions between the adjacent layers of graphite is necessary.

#### 4.7.3. Liquid-phase exfoliation from expanded graphite (CUI)

**Figure 10:**
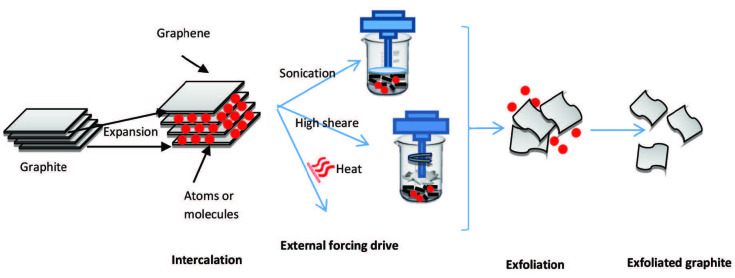
Intercalation and exfoliation.

Graphite intercalation compounds (GICs) are formed by the insertion of atomic or molecular layers of a different chemical species (such as alkali metal atoms or acid molecules, as atoms or molecules) called the intercalant between host graphite sheets [103], see Figure 10. The graphite intercalation compound (GICs), also known as the expanded graphite, is a synthesized intercalation compound of graphite that expands or exfoliates when heated. The intercalation increases the distance between the graphite layers. In pristine graphite, the distance between the layers is typically 0.34 nm, while in a potassium-GIC, the sheet distance has been measured to about 0.53 nm [103]. Because of the increased distance between the graphite layers in the GIC, the van der Waals attraction between the layers was weakened. Thus, the layers can be more easily separated to acquire the desired graphene [104]. The number of layers separated by an intercalant layer is defined as staging. The stage index n denotes the number of graphite layers between adjacent intercalate layers of structural ordering. Fully intercalated graphite will be a stage 1 GIC [105]. The graphite intercalation compounds are of particular physical interest because they have a relatively high structural ordering degree. Although graphene is a material with a single carbon layer, the definition has been broadened in recent years, and the term ‘’few-layer graphene’’ is often used. If the number of carbon layers is 10 or more, the piece is known as a graphite nanoplatelet (GNP), also known as an exfoliated graphite nanoplatelet (GNP) or a graphite nanosheet (GNS) [106].

Güler et al. 2016 [107] prepared multilayer graphene by mixing hexagonal graphite (HG) with an acidic mixture to form graphite- intercalated compound (GIC). The GIC is then heated at a temperature between 800–900 ºC to form expanded graphite (EG), followed by dispersion in an organic solvent through ultrasonication.

The exfoliated graphite exhibited excellent properties of flexibility, lubricity, and adsorption. The exfoliated graphite has many applications in various fields, such as absorption of spilled oil in water [108,109], sealing material [110], fire retardant [111], and electrode [112]. 

#### 4.7.4. Liquid-phase exfoliation by organic solvent

Graphite can be exfoliated into single-layer graphene (SLG) and few-layer graphene (FLG) in a solvent having a surface tension of about 40 mJ/m^2^ [33,91]. Graphite’s surface energy is the minimum energy per unit area sufficient for the van der Waals force when two sheets are peeling apart. Graphene and solvent surface energies have to be very close for a successful exfoliation process. The enthalpy of mixing will be reduced, and the exfoliation will increase. Liquids with surface tensions of about 30–40 mJ/m^2^ are typically good solvent candidates for graphene exfoliation, such as N-methyl-2-pyrrolidone (NMP 40 mJ/m^2^) [33], N, N-dimethylformamide (DMF 37.1 mJ/m^2^) [113].

Sonication-assisted liquid-phase exfoliation of graphite to give Graphene was first reported by Hernandez in 2008 [33]. In their work, graphite powder was dispersed in a specified organic solvent, such as N, N-dimethylformamide (DMF), and N-methyl pyrrolidone (NMP), followed by sonication and centrifugation. The LPE yield is estimated by measuring graphene concentration in the dispersion using UV-Vis spectroscopy and exploiting the Beer–Lambert law. The degree of exfoliation was determined by analyzing the number of layers in a graphene flake using TEM, AFM, and/or Raman spectroscopy. Defect-free monolayer graphene, bi-layer, and multilayer graphene are obtained. The solubility of graphene in the dispersion was 0.01 mg/mL after 30 min sonication, centrifugation at 500 rpm for 90 min. The number fraction of monolayer graphene in NMP dispersions was estimated at 28%. It was found that NMP gives the best thermodynamic stabilization due to the good matching of graphite –solvent surface energies. The most important criterion for successful graphite exfoliation is the good matching of solvent surface energies with graphene. The best solvent is that with surface energies close to the graphene surface energy [114–116].

Graphite consists of many graphene layers joined together by van der Waals attractions between the adjacent layers. Successful exfoliation of graphite requires the overcoming of the van der Waals attractions forces between the adjacent layers. When graphite is immersed in a liquid, the interfacial tension between graphite and solvents should be equal to the graphene-graphene interaction energy [117]. A very close graphite- solvent surface energies resulted in small mixing enthalpy, and the exfoliation occurs more easily [118]. Good solvents are used to disperse graphene minimizing the interfacial tension between the solvent and graphene flakes. 

The liquid’s surface tension is the force required to resist an external force due to liquid molecules’ cohesive nature. If the interfacial tension between graphitic flakes and the solvent is high, there is poor dispersibility of the solvent’s graphitic flakes [114]. The flakes tend to adhere to each other due to the flakes’ cohesion force and hindering their dispersion in the solvent [119]. Hernandez et al. [33,91] reported that solvent with surface tension 40 mN/m is the best solvents for dispersing graphene and graphitic flakes. This will minimize the interfacial tension between the solvent and graphene [119]. 

It has to be noted that direct LPE in organic solvents like NMP normally produces graphene flakes with lateral dimensions typically < 1 μm, which are too small for applications such as mechanical reinforcement in composites.

The solvent molecule-graphene interactions were investigated by Liu et al. [120] using electrophoresis and zeta potential measurements. The charge transfer between the graphene and solvent molecules is responsible for the graphene’s surface charge. Graphene can be positively or negatively charged in the dispersion exhibiting different zeta potential values depending on the different donor and acceptor numbers of a solvent. The electrostatic repulsion of charged graphene sheets will ease the stable dispersions. 

The main disadvantages of using a solvent with a surface tension of 30–40 mJ/m^2^ are the solvents’ toxicity. NMP organic liquid is an eye irritant and also toxic to the reproductive organs [121]. DMF may have toxic effects on multiple organs [122].

The other disadvantages of this method are the low stability, low single-layer content (0.01 mg/mL), and the solvents’ high boiling points make it very difficult to remove the solvent after exfoliation [33]. 

Therefore, the search for additional solvents is highly recommended to overcome the low yield problem associated with graphene dispersions in organic solvents.

#### 4.7.5. Liquid-phase exfoliation by ionic liquids 

Utilizing ionic liquids (ILs) in graphite exfoliation is considered green chemistry due to it is nontoxic, stable chemically, no need for vapor pressure, recyclable by distillation or ion change. Exfoliation by ionic liquids is also considered a one-step procedure (direct exfoliation). Ionic liquids give graphene unique properties, unlike chemical modification that destroys the graphene electronic structure. Ion liquids are completely organic or partially inorganic salts with a melting point below (100 °C). The most significant factor in this technique is the surface tension of ILs, which has to be very close to that of graphene [123,124].

Fukushima et al. were the first to use ILs to untangle carbon nanotubes using imidazolium-based ILs as solvents assuming interactions between the positive charge of the imidazolium rings and p electrons of the nanotube [125]. Liu et al. prepared graphene nanosheets using imidazolium ILs assisted electrochemical synthesis [126]. Zhou et al. incorporated polymerized ionic liquid with 1-butyl-3-methylimidazolium hexafluorophosphate to disperse graphene. Nonoxidized few-layer graphene highly concentrated and stable colloidal (0.95 mg/mL) was prepared by ultrasonication of natural graphite flakes in this ionic liquid [127]. Nuvoli et al. prepared high concentration few-layer graphene sheets using LPE by grounding and then sonication of graphite in 1-hexyl-3-methylimidazolium hexafluorophosphate (HMIH) as an ionic liquid. The graphene concentration (up to 5.33 mg/ mL) is obtained. Ionic liquids can be useful when green chemistry and/or a nonvolatile, stable solvent medium is required [124].

In 2014, Shang et al. prepared large quantities of solvent-free graphene nanosheets and nanodots with low oxygen content have by mechanical grinding exfoliation of natural graphite in a small quantity of ionic liquids (1-Butyl-3-methylimidazolium hexafluorophosphate, BMIMPF6) [128]. Electrochemical exfoliation of graphite rods was used to synthesis carbon dots (CDs) by dissolved cheap salts (NaCl and KCl) in distilled water as an electrolyte [129]. Generally, exfoliation using ionic liquids has been associated with other techniques as electrochemical exfoliation, mechanical grinding, or sonication to be more producible.

#### 4.7.6. Liquid-phase exfoliation by surfactants

Liquid phase exfoliation by a surfactant is considered an ideal method to prepare high stable concentrated graphene dispersion despite the surfactant’s defects [130]. Six surfactants were utilized involving ionic and nonionic: sodium deoxycholate SDBS, sodium dodecyl sulfate HTAB, Ssodium dodecyl benzenesulfonate SDS, hexadecyltrimethylammonium bromide, and Triton X-100 and Tween 80. The theory explains the different ionic and nonionic surfactants’ different mechanisms in stabilizing graphene dispersions for colloidal stability [130]. Commercially, there are around 20–30 available surfactants that have been used to exfoliate graphite. The main types of surfactants are cationic, anionic, and nonionic surfactants. Sodium dodecyl sulfate (SDS) is an anionic surfactant with a small molecule with a hydrophobic tail and a polar head group [131]. In addition to previous classes, pluronic and tetronic block copolymers were used to produce graphene dispersions with concentrations exceeding 0.07 mg mL-1 via sonication and centrifugation [132].

Ionic surfactants such as cetyltrimethylammonium bromide (CTAB) [133], sodium dodecylbenzene sulfonate (SDBS) [33], sodium cholate (SDOC), and other surfactants [134] were used to exfoliate graphite flakes. Ultrasound treatment of graphite dispersion in N-methyl pyrrolidone solution yields a dispersion with a concentration of 2.21 mg/mL [135]. Graphene dispersed in a monomer (tetraethylene glycol diacrylate), and the latter’s subsequent polymerization yields a dispersion of 9.45 mg/mL [136]. For graphene dispersions in two organosilanes, 3-glycidoxypropyl trimethoxysilane (GPTMS) and phenyl triethoxysilane (PhTES), the concentrations were 0.66 and 8.00 mg/mL for PhTES and GPTMS, respectively [137]. Guardia et al. examined the differences between ionic and nonionic surfactants and showed nonionic surfactants’ dominance [138]. Niu et al. reported a low-cost and environmentally friendly method to directly exfoliate graphite powders into few-layer graphene sheets using inorganic salts to expand graphite for easy exfoliation by low-power ultrasonication [139]. The addition of ethanol to water/surfactant solutions can lead to a high concentration (∼0.46 mg/ml) of graphene dispersions. About 10 wt% of ethanol addition for ionic and nonionic surfactants was found to enhance the exfoliation efficiency and the graphene concentration three times. This enhancement is due to decreasing mixing enthalpy and enhancing the stability of surfactants [140,141].

A simple and inexpensive green route for large-scale production of exfoliated graphene dispersions using direct sonication of graphene and aqueous solution of perylene tetracarboxylate (PTCA) aromatic semiconducting surfactant. This produced a high yield of single and few-layer graphene sheets with minimal basal plane defects [142]. A surfactant-assisted ultrasonic exfoliation of mildly oxidized graphite has been successfully demonstrated using cationic surfactants and one anionic surfactant. The surfactant charge was found to be a significant factor in affecting the exfoliation efficiency [143].

#### 4.7.7. Liquid-phase exfoliation by low boiling solvents

One of graphene’s solvent exfoliation problems is that the good solvents tend to have high boiling points that present problems for flake deposition, so they also are difficult to remove. Low boiling point solvents such as chloroform, isopropanol, and 1-propanol were explored by many researchers for LPE production of graphene because of the removal difficulty of high boiling solvents like DMF NMP, etc. [141,144,145]. Catheline et al. prepared individualized graphene flakes as single and double layers with one micron as average lateral size by dissolving the graphite intercalation compounds KC8 in low boiling points ether solvents such as methyl-tetrahydrofuran (Me-THF, bp= 77 °C), tetrahydrofuran (THF, bp = 65 °C), and cyclopentyl methyl ether (CPME, bp = 106 °C). This method is expected to significantly benefit composites and thinner film preparation due to high dispersity, stability, or double graphene levels [146].

### 4.8. Electrochemical exfoliation of graphite 

**Figure 11:**
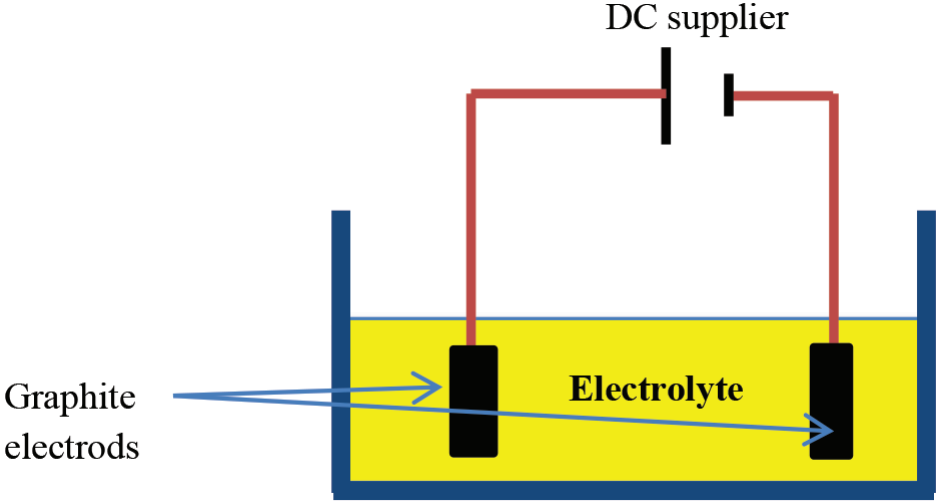
Electrochemical exfoliation [147].

When a voltage is applied in a conductive solution for the graphite electrodes, the graphite electrodes can corrode and produce the solvent-functioning graphite nanosheets (see Figure 11) [147]. The use of electrochemical exfoliation of graphite anode to prepare graphene was first reported by [126] using a mixed solution containing ionic liquid and water as the electrolyte. Structure with up to 10 graphene layers is called few-layer graphene, while the structure with more than 10, and less than 100 graphene layers is called thin-film graphite [148]. Liu and his group [149] believed that electrochemical exfoliation would play more critical roles in synthesizing graphene materials, but many exciting challenges are still waiting to be explored. These challenges include a better understanding of graphite exfoliation reaction mechanism, parameters that affect large-scale graphene production, such as complex fluid mixing, heat, and mass transport. Finally, the obtained graphene material is suitable for many applications with unique features. For example, in membrane applications, graphene materials must behave tailored size, interlayer spacing, and porosity. Su et al. prepared high-quality graphene sheets by electrochemical exfoliation of graphite. The electrochemical cell consists of graphite anode and platinum cathode in a 0.5M of H_2_SO_4_ solution. The exfoliated graphene sheets exhibit lateral size up to 30 μm [150]. Although this method is easy to implement and prepare graphene, the obtained graphene still has many oxygen functional groups and structural defects due to the oxidation reactions at the graphite anode. 

Morales et al. used an electrochemical route to produce few-layer graphene from electrochemically-produced graphite intercalation compounds in aqueous perchloric acid. Anodic intercalation is more efficient in terms of time. To prevent the formation of graphite oxide, cathodic pretreatment is preferred. Experimentally, it was found that using electrochemical-potential control is very promising to obtain graphene with different degrees of oxidation [151].

An electrochemical route for producing graphene sheets by electrochemical intercalated sodium dodecyl sulfate (SDS) into graphite followed by electrochemical exfoliation of an SDS-intercalated graphite electrode was reported by Alıoğlu et al. [152]. The structural order, size, and a number of layers of synthesized graphene sheets depend on the potential value for SDS intercalation into graphite. Graphene sheets with the highest structural order and lowest number of layers can be obtained using relatively high intercalation potentials. The average size of 500 nm and a monolayer of graphene sheets were prepared at high SDS intercalation potentials into graphite. After successive electrochemical intercalation and exfoliation processes, UV–vis spectra of graphene/SDS suspensions showed a large amount of the reduced form of graphene flakes was obtained [152].

Anodic exfoliation of graphite in (NH_4_)_2_SO_4_ aqueous solution was reported by Chen and Xue [153]. The proposed electrochemical exfoliation mechanism showed that SO_4_^2−^ and H_2_O could be intercalated into those graphite sheets. Monolayer and few-layer graphene were obtained due to the SO_2_ and O_2_ gases formed within graphite sheets. The exfoliated colloidal graphene can be perfectly stabilized in DMF solvent for more than 1 week. The colloidal graphene can show promising applications in flexible printed electronics and energy storage devices [153]. 

A nonoxidative production route to few-layer graphene via the electrochemical intercalation of tetraalkylammonium cations into pristine graphite using two types of graphite was presented by Cooper et al. [154]. Highly orientated pyrolytic graphite (HOPG) and a graphite rod were employed in the 3-electrode system, consisting of a platinum wire counter electrode and silver wire (with glass frit) or platinum wire as the pseudo- reference electrode. Highly orientated pyrolytic graphite (HOPG) gives greater advantages in terms of the exfoliate size, but there are difficulties in the electrode set up and require the use of sonication. The use of graphite rod electrode does not require ultrasonication or any secondary physical processing of the resulting dispersion. Few-layer graphene flakes with 2 nm thickness are formed directly with flake diameters from 100–200 nm [154]. 

Seville et al. [155] presented a facile and environmentally friendly synthetic strategy to produce stable and easily processable Graphene dispersions in water. This method is an alternative to classical chemical exfoliation methods such as the Hummers method, which is more complex, dangerous, and harmful. The new strategy is based on the electrochemical exfoliation of graphite. It includes three simple steps: anodic exfoliation of graphite in (NH_4_)_2_SO_4_, followed by sonication to separate the oxidized graphene sheets, and then reduce oxidized graphene to graphene. With this strategy, it is possible to convert around 30 wt % of the initial graphite into graphene with short processing times and obtain high yields. The graphene sheets are well dispersed in water, have a lateral size of about 0.5–1 μm, and contain only a few graphene layers, most of which are bilayer sheets [155]. 

A. Öztürk and M. Alanyalıoğlu prepared graphene sheets using two-step electrochemical processes in a two-electrode cell system containing 0.1 M sodium dodecyl sulfate (SDS). Using different intercalation potential values of 1, 3, 5, and 7 V, the SDS was intercalated into graphite anode electrode. Then, the SDS-intercalated graphite electrode was exfoliated in the same medium by acting as a cathode. Stable graphene dispersions were obtained after these two electrochemical steps. A new glassy carbon electrode (GCE) was modified with graphite, graphene/GCE. This new graphene/GCE electrode has good electrocatalytic activity and has been used for amperometric determination of nitrite in both standard laboratory and real samples [156]. 

The synthesis of GO by the electrochemical method is utilized recently. This involves the electrochemical conversion of a graphite electrode (as an anode) into graphene under high potential, followed by its chemical oxidation. The graphite anode is inserted in the electrolyte solution, i.e. H_2_SO_4_, and a high voltage DC is applied, which exfoliates graphite into graphene. Thus, graphene is collected and then oxidized into GO by chemical oxidation using MHM [157]. The process is easy and manageable for large-scale production. The quality of GO prepared can be controlled by sweeping the voltage and type of electrolyte [97].

Generally, the electrolyte for this process can be ionic liquids [126], aqueous acids such as H_2_SO_4_ [158], and aqueous solutions of different inorganic salts ((NH_4_)_2_SO_4_, Na_2_SO_4_, K_2_SO_4_, etc.) [159]. A high yield of FLG (>70%) was prepared by electrochemical charging of graphite electrodes in a Li+/propylene carbonate electrolyte. This high yield of FLG obtained from the graphite electrode is significantly higher than that produced by most current liquid-phase exfoliation methods [160]. 

The electrolyte plays a crucially important role in the yield and quality of electrochemically synthesized Graphene. A lower amount of graphene is produced by exfoliation in ionic liquids with a small graphene’s small lateral size. The electronic properties of the produced graphene are distorted due to functionalization with ionic liquids [126]. However, high-quality graphene with a large lateral size is produced via exfoliation in acidic electrolytes. Nevertheless, over-oxidation of graphite by acidic electrolytes leading to the presence of oxygen-containing functional groups [150,158,161].

A facile, mild, and environmentally-friendly approach for the efficient electrochemical exfoliation of graphite was developed using a sodium hydroxide/hydrogen peroxide/water (NaOH/H_2_O_2_/H_2_O) system that can produce high-quality, anodic few-layer graphene nanosheets in 95% yield at ambient reaction conditions [162].

Exfoliated graphene oxide/multilayer-graphene (GO-MF) flakes were obtained by electrochemical exfoliation of industrial graphite using tungsten (99.5% W) as the counter electrode and graphite as the working electrode [163]. The analytical techniques and XRD results showed 67 wt.% graphene oxide (GO) and 33 wt.% multilayer-graphene (MG). The mechanism of electrochemical exfoliation of graphite in ionic liquid solution involves (i) anodic oxidation of water, (ii) hydroxyl and oxygen radicals’ generation, which attacks the edges of the various graphite sheets (iii) intercalation by ionic liquid anions between graphite layers, forming the graphite-intercalation-compounds (GICs), and (iv) oxidative cleavage and precipitation of the GICs [164]. Lin et al. showed the electrochemically exfoliated graphene flakes have higher crystallinity and fewer defects than graphene flakes prepared by liquid phase exfoliation [165].

Most of the graphene synthesis through electrochemical exfoliation has used acidic electrolytes typified by H_2_SO_4_. The size of SO_4_^2-^ ion in solution is ~4.60 Å, which is about ~1.37 times higher than the interlayer spacing of 3.35 Å between graphitic layers in graphite. These SO_4_^2- ^ions under applied voltage intercalate between graphitic layers, and they push the graphitic layers farther apart, making the spacing greater than 3.35 Å. This leads to graphite exfoliation because of the weakening of Van der Waals bonding [165,166].

In electrochemical exfoliation with alkaline electrolyte, both OH ions (have a size of ~0.958Å) and the hydrated OH ions (have a size of ~2.503 Å) are smaller than the graphitic interlayer spacing of 3.35 Å. The electrolyte with hydrated OH- ions (size ~2.503 Å) behave as polar, and due to the applied electric field, they enter the graphitic layers. These ions will polarize the graphitic layers located above and below, and the hydrated OH- ions will get coupled with the graphitic layers with electrostatic interaction. Under optimum electric force, the hydrated ions will pull the graphitic layers above and below out of the graphitic stack. The graphene-hydrated OH- configuration forming bilayer Graphene will thus get formed and float in the alkaline electrolytic solution [166]. 

The three main advantages of an electrochemical method of graphene production are: Firstly, it is used to obtain high-quality graphene with no use of hazardous oxidizing materials. Secondly, the method is relatively simple such that a scale-up to industrial production levels could be comparatively straightforward. Moreover, thirdly, hazardous chemicals and the lack of necessity for temperature control and makes the electrochemical production of GO more environmentally friendly [126,148,150,167]. 

## 5. Exfoliation methods’ summary 

Table 1 summarizes the previous and recent representative studies on the synthesis of graphene by exfoliation from different carbon sources and reacting media.

**Table 1:** Table 1. Summary of graphite exfoliation methods.

#	Carbon source	Methods	Media	Results		YearRef.
	Flake graphite powder	Oxidation by acids	Like NaNO3+KMnO4, sulphonitric acid….	graphene oxide suspension	1898193719581977	Staudenmaier [54], Hofmann and König [55], (Hummers and Offeman [56] and Berger and Maire [168]
	Flake graphite powder	Oxidation +heating up to 1000 °C	H2SO4, FeCl3, Na-tetrahydrofuran(THF), K-THF and Co-THF	different intercalation compounds (GICs)	1991	Yoshida et al. [169]
	Natural graphite powder and plates (HOPG)	chemical oxidation(calorimetric and potentiometric methods are)	by gaseous oxidizers (Cl2,O2, SO2)	intercalation compound (GIC)	1996	Avdeev et al. [170]
	Natural graphite flakes	electrochemicalprocess	formic acid solution	(HCOOH-GIC) the specific surface area of 20 to 50 m2/gexpansion vol. reaches 150–300mL/g	1997	Kang et al. [171]
	Graphite flake	direct reaction with gases + heating	gaseous SO3	intercalation compounds(GIC) Vol% incresing 220 times and 400 times density 0.0053-0.01 g/cm3, pore size 10-170 µm,	2002	Lee 2002 [172]
	Graphite	Microwave-assisted Intercalation and exfoliation	intercalated with a mixture of nitric and sulfuric acid, exfoliated by ethanol exfoliation	nanoplatelets with thicknesses 2–10 nm approximately 30 layers.	2005	Viculis et al. [173]
	Graphite oxide	reduction of exfoliated graphite oxide by amphiphilic polymer	Poly sodium 4-styrene sulfonate PSS	1 mg/mL graphitic nanoplatelets	2006	Stankovich et al. [97]
	Natural flake graphite	Staudenmaier method to produce GO, then thermal expansion of graphite oxide	nitric acid +sulfuric acid + potassium chlorate and hydrochloric acid	functionalized graphene sheets	2007	Mcallister et al. [67]
	Natural graphite	Hummers method to produce GO, then reduced by Hydrazine	Hydrazine hydrate (1.00	graphene-based nanosheets	2007	Stankovich et al. [36]
	Graphite	Ionic liquid-assisted electrochemical approach (one step)	1-octyl-3- methyl-imidazolium hexafluorophosphate ([C8mim]þ[PF6]--	Graphen sheet with 1mg/mL and average thickness 1.1nm	2008	Liu et al. [126]
	Commercial expandable-graphite	Exfoliation by heating to 1000 °C, grounding, re-intercalatewith different substances	Oleum+TBA+DMF	high-quality graphene sheet	2008	Li et al. [35]
	Graphene oxide	the chemical reaction of GO	GO water suspension with KOH and then with hydrazine	7 mg/mL concentration, the thickness of 6 Å, lateral size ranges of several hundreds of nanometers to a few micrometers	2008	Park et al. [99]
	Graphite	Aqueous-phase exfoliation	sonication in polyvinylpyrrolidone biopolymee+water	Water-soluble graphenes single layers -without oxidation or destruction - concentration of 0.15–0.2 mg/mL-average thickness of these flakes was less than 1 nm (0.7–0.9 nm)	2009	Bourlinos et al. [174]
	Graphite	Oxidation by Hummers method and Intercalation by ionic liquid	Tetrabutylammonium TBA+DMF	Graphene	2009	Ang et al. [98] [98]
	Graphite	Cationic surfactant mediated exfoliation+sonication	cetyltrimethylammonium bromide CTAB + acetic acid	graphene flakefew layered graphene nanoflakes. The average thickness of the flakes was found to be 1.18 nm	2009	Vadukumpully et al. [133]
	Graphene	covalently functionalized with ionicliquid	amine-terminated ionic liquid	0.25–0.5 mg/mL polydisperse graphene sheets	2009	Yang et al. [175]
	Natural graphite flakes	ultrasonication in ionic liquid	ionic liquid [bmim][PF6] stabilized by an ionic liquid polymer (PVP+PSS).	highly concentrated and stable suspension (0.95 mg/mL) of nonoxidized few-sheets graphene	2009	Zhou et al. [127]
	Graphite powder	Liquid phase technique in surfactant/water	Sodium dodecylbenzene sulphonate (SDBS)	More than 40% of these flakes had <5 layers with ~3% of flakes consisting of monolayers.	2009	Loyta et al. [34]
	Graphite powder	liquid-phase technique in aromatic solvents	C6F6, C6F5CF3, C6F5CN and C5F5N	0.1 mg/mL admixture of suspended graphenes (10%–15%), nanosheets, and graphite particles	2009	Bourlinos et al. [176]
	Graphite	Staudenmaier method, then grinding and functionalization in ionic liquid	functionalization of graphene using aryl-diazonium salts and (OMIBF4) in ILs	Functionalized graphene sheets	2009	Jin et al. [177]
	Natural graphite	Expanded with acids, then decomposed by heating	H2SO4 and H2O2	Graphene sheets of high quality	2009	Gue et al. [178]
	Graphene	via a modified Brodie method to prepare GO, and then reduced by reducing agent	reducing agent, NaBH4 and N2H4	------	2009	Shin et al. [70]
	Graphite	Solvothermal method(heated in a sealed reactor vessel at 170 ˚C)	a mixture of sulfuric andnitric acid +oleyl amine was 1C for 72 h	the large size of graphene flakes up to 300 mm2/gm with a concentration of 0.15 mg/mL.	2010	Zheng et al. [179]
	Graphene oxide	Solvothermal reduction of GO	in organic solvent (NMP)	0.93 nm sheet thick	2010	Dubin et al. [101]
	Graphite	Electrochemical exfoliation of into aqueous solutions	HBr, HCl, HNO3, and H2SO4,Then disperse in DMF	graphene sheets thickness lower than 3 nm, the lateral size of these from 1 to 40 μm	2011	Su et al. [150]
	Graphite	grounding and sonicating graphite in liquid ion	1-hexyl-3-methyl- imidazolium hexafluorophosphate (HMIH)	5.33 mg/mL of graphene with few layers	2011	Nuvoli et al. [124]
	Graphite	Exfoliation in different surfactants	-Ionic (Pluronic, Tween 80, Brij 700, Gum arabic from, Triton X-100 Tween 85, and Brij30)-Nonionic (PVP, DBDM, PSS, CHAPS,DOC, SDBS, PBA, SDS,TDOCand HTAB)	High graphene concentrations, up to 1 mg/mL, single- and few-layer graphene platelets	2011	Guardia et al. [138]
	Graphene	Exfoliation in low boiling point solvents	Acetone, isopropanol, chloroform cyclohexanone, N-methyl pyrrolidone, and dimethylformamide.	0.5mg/L graphene flakes of∼1 μm length and with a thickness of less than 10 layers	2011	Neill et al.[141]
	Graphene oxide	Fe reduction of exfoliated graphite oxide	GO prepared by hummers methods, then reduced by Fe powder and a few amount of HCl.	2–10 stacked graphene nanosheets	2011	Fan et al. [102]
	Graphite	Electrochemical Expansion	Propylene Carbonate Electrolyte	High-yield (>70%) of few-layer graphene flakes average thickness <5 layers	2011	Wang et al. [160]
	Natural graphite flakes	sonication and centrifugation of graphite with nonionic, amphiphilic block copolymers	Pluronic and Tetronic block copolymers	graphene dispersions exceeding 0.07 mg/mL	2011	Seo et al. [132]
	Natural graphite	mechanical grinding exfoliation in a small quantity of ionic liquids	ionic liquids (1-Butyl-3- methylimidazolium hexafluorophosphate, BMIMPF6)	graphene nanosheets and nanodots. The sheets are only two to five layers thick. The graphene nanodots have diameters in the range of 9–29 nm and heights in the range of 1–16 nm	2012	Shang et al. [128]
	Flake graphite	wet chemical bulk functionalization rout	Reduction by a liquid alloy of sodium and potassium+DMF and then intercalated by K ion, functionalized by diazonium salts BPD and SPD.	Functionalized graphene sheet with heights of 2–6 nm. 3 nm averaged at flat regions	2011	Englert et al. [37]
	Natural graphite flakes	organic solvent exfoliation+sonication	BzCl, DCB, GBL, CYC, DMF, NMP, DMA, TMU, PYR, and BzAm.	unfunctionalized graphene, positively or negatively charged when it was dispersed in organic solvents in an electrical field and zeta potential	2012	Liu and Wang [120]
	Synthetic and natural graphite	Surfactant-assisted exfoliation technique, sonication with continuous addition of surfactants.	Cationic, anionic, nononic surfactants C12H33N(CH3)3BrC14H33N(CH3)3BrC16H33N(CH3)3BrC12H25OSO3NaF127F108	Very high concentration15 mg/mL	2012	Notley [180]
	Pencil core	Electrochemical exfoliation	Exfoliation in (H2SO4 orH3PO4) and reduced with KOH	1-5 layers, 5.45 nm thickness, 2-micron lateral size with a sharp edge and flat surface	2013	Liu et al. [161]
	Vitreous carbon foam sheet	Electrochemical Method	in NaOH + KCl	nanoscale GO flakes with an average length of 5nm.	2013	Parker et al. [167]
	Natural graphite powder	Oxidation+ stirring(improved Hummers method without using NaNO3)	H2SO4+KMnO4+H2O2 then washed by 1:10 HCl	GO, yields 92% - 96%	2013	Chen et al. [58]
	Natural graphite	Intercalated by acid then irradiated by microwave	hydrogen peroxide, nitric acid, and acetic acid.	thin graphene-like nanosheets 1 to 3 nm	2013	Xiu-Yun [181]
	Graphite powder	Modified Hummers method and then reduced by hot pressing under the vacuum up to 1500 °C	Hummers acids	The graphene sheets with few defect and oxygen content	2013	Zhang et al. [66]
	Graphite flake	electrochemical exfoliation	H2SO4 solutions	high yield (>80%) of one- to three-layer graphene flake with sheet size(∼10 μm), low oxygen content (7.5%), and (∼1 mg/mL )	2013	Parvez et al. [158]
	Chinese natural large flake graphite	electrochemical exfoliation	Diluted H2SO4 50%	100–500 μm particle size of GIC-bisulfate	2013	Asghar et al. [182]
	Graphite powder	surfactants LPE (H2O+surfactant )+10% ethanol to enhance CG .	Tween 80Triton X-100 sodium deoxycholate (SDOC)sodium dodecyl sulfate (SDS)	CG 0.46 mg/mL three times >without ethanol average area 52.12 mm2thickness of 1-3 nm	2014	Wang et al. [140]
	Graphite powder	Liquid Exfoliation of Graphite (Oxidizing and Ultrasonicating)	(KMnO4+H3PO4+H2SO4) then H2O2	GO, 80% of the sheets are single or few layers with lateral size 50–100 nm, CG (0.1 mg/mL)	2014	Shia et al. [183]
	Graphite	Electrochemical exfoliation of into aqueous solutions	inorganic salts (NH4)2SO4, Na2SO4,K2SO4, etc.).	graphene with a high yield (>85%, ≤3 layers), large lateral size (up to 44 μm), low oxidation degree (a C/O ratio of 17.2)	2014	Parvez et al. [159]
	Graphite	liquid-phase exfoliation (LPE) process, low power sonication for long times	sodium cholate [166] , N- methyl-2-pyrrolidone (NMP) [167][168]	homogenous dispersions of unfunctionalized and non-oxidized graphene nanosheets, defect-free, few-layer-thick	2008,2010, 2011, 2014	Hernandez et al. [33]Loyta et al. [184], Khan et al.[185], Ciesielski et al. [186]
	Graphite	Liquid phase exfoliation by organic solvent	Oxidation by acids (H2 SO4+HNO3)DMF solvent	Graphene dispersion	2016	Güler et al. [107]
	Natural graphite flake	Liquid phase exfoliation by different surfactants (ionic and nonionic)	SDOC, SDBS, SDS,HTAB, Tween 80 and Triton X-100	Graphene dispersion	2016	Wang et al. [130]
	Graphite powder	Modified Hummer methods to prepare Go then reduced by plant extact.	(NaNO3+ KMnO4+ H2SO4+HCl) as oxidizers, and black tea as a reducing agent.	graphene dispersion	2017	Moosa and Jaafar [77]
	Graphite	Surfactant assisted liquid phase exfoliation	Perylene tetracarboxylate surfactant	graphene nanosheets in aqueous/organic polar solvents	2017	Narayan et al. [142]
	Graphite	Oxidation by acids and	HClO4 and KH2PO4 as the inserting agent and KMnO4	Sulfur-free (GIC)	2017	He et al. [187]
	Helical CNTs	Unzipping technique using sonochemical approach	H2SO4+HNO3	Graphene nanoribbon	2018	Abed [188]
	Clip pressed flake graphite electrode	Electrochemical exfoliation (special design electrod)	M (NH4)2SO4	Graphene with high yield (65%) and large lateral size (>30 μm)	2018	Achee et al. [189]
	Artificial graphite	Modified Hummers method	Different surfactants DDACl, DDBAC, DTAC and SL	Highe yield of few layers GO	2019	Hu et al. [143]
	Fluorinated graphite (FGi)	chemical reaction of FGi with hydrazine hydrate	Hydrazine hydrate and absolute ethanoi	monocrystalline graphene	2019	Cheng and Meng [89]
	Natural graphite	LPE by low-boiling point solvent assisted ball milling	simple salt K2CO3	monolayer graphene, high yield (more than 10%) after a few minutes of sonication	2019	Arao et al. [190]
	Graphite rods	salt-assisted electrochemical exfoliation	NaCl water solution	Carbon dots	2019	Li et al. [129]

## 6. Summary and discussion

This paper reviewed the most common graphene fabrication methods. It involves the concept and technical development and modification in each process since its first inception in 1898. This review focuses on graphite exfoliation and its progress because it is considered the most popular method for graphene production with high yield and different properties. Most of the scientific works are interested in the mechanism of exfoliation, targeting how to get more yield, low O content, fewer layers, and large lateral size, best properties, low cost, more eco-friendly, and with fewer steps. 

Many excellent reviews have been published on the production and processing of graphene, including Cai et al. [13], Bonaccorso et al. [119], and Chung et al. [104].

Cai et al. [13] presented an excellent general review on graphite exfoliation methods and discussed the bulk production of graphene via exfoliation, focusing on the exfoliation techniques and yields. Different precursors are used for exfoliation such as graphite oxide, pristine graphite, graphite intercalation compounds, and expanded graphite, but the yield of monolayer graphene is still relatively low. Furthermore, another drawback to the exfoliation of graphene sheets in solvents is the aggregation of these sheets due to Van der Waals attraction, which compromises the exfoliation efforts. Chung et al. [104] gave an excellent general review on exfoliated graphite (EG), which is graphite that has a degree of separation of a substantial portion of the carbon layers in the graphite. Graphite nanoplatelet (GNP) is commonly prepared by mechanical agitation of EG. Bonaccorso et al. [119] reviewed graphene as a new class of 2D materials derived from layered bulk crystals. They emphasize the requirement of graphene application and the development of industrial-scale, reliable, inexpensive production processes.
